# Eosinophilic Fasciitis in a 68-Year-Old Female

**DOI:** 10.7759/cureus.53908

**Published:** 2024-02-09

**Authors:** Swetha Chittipolu, Jennifer L Kennard, Ludmila Nahar

**Affiliations:** 1 Internal Medicine, North Mississippi Health Services, Tupelo, USA; 2 Rheumatology, North Mississippi Health Services, Tupelo, USA

**Keywords:** steroids, peripheral eosinophilia, tornado, absolute eosinophil count, eosinophilic fasciitis

## Abstract

Eosinophilic fasciitis (EF) is an uncommon disorder of unknown etiology and poorly understood pathogenesis. In this report, we present a case of a 68-year-old female presented with a rapidly progressing skin tightening condition in her extremities associated with eosinophilia. Four months prior, the patient's initial complaint was skin sensitivity in the legs and forearms. Over time, this led to severe skin tightening, edema, and decreased range of motion. Clinical examination showed tightening of the skin over the anterior forearms, posterior knees, and calves without sclerodactyly or Raynaud's phenomenon. Laboratory investigations showed eosinophilia, elevated antinuclear antibody titer, and negative rheumatoid factor. This presentation raised suspicion of EF, and biopsy results showed scattered lymphocytic infiltrate involving associated fibrous tissue and perivascular lymphocytic inflammation that involved vessel walls. She was treated with low-dose steroids due to her diabetes but the stiffness continued. She was started with immunomodulators methotrexate, which showed improvement in symptoms, including softening in her arm tissues.

## Introduction

Eosinophilic fasciitis (EF) was first reported in 1974 by Shulman [1]. EF is a rare condition characterized by symmetric bilateral induration of the skin and deeper peri muscular fascial planes. The onset is acute, findings include erythema, swelling, and induration of the extremities that is accompanied by peripheral eosinophilia. Skin changes typically spare the fingers, hands, and feet [2]. Diagnosis is based on full-thickness skin-muscle biopsy. It is crucial to differentiate it from systemic sclerosis due to differences in clinical presentation and management. Glucocorticoids are the mainstay of treatment, and immunosuppressive or immunomodulatory agents are required to obtain a therapeutic response [3].

## Case presentation

A 68-year-old female with a history of hypertension, obstructive sleep apnea, diabetes mellitus, and coronary artery diseases, presented with skin tightening that began as skin sensitivity on her legs and forearms four months ago. Her symptoms rapidly progressed, involving the anterior forearms, posterior knees, and calves. The tightening limited her range of motion, particularly in the arms and legs along with wrists, leading to difficulties in hand closure and gripping items. Swelling was noted in the legs, knees, and ankles. She was started on prednisone 15 mg daily (not a higher dose due to diabetes). This helped with swelling in knees and ankles but did not alleviate stiffness and associated aching pain. She denied symptoms of Raynaud's phenomenon and dysphagia.

Laboratory investigations revealed eosinophilia, with absolute eosinophil counts (AEC) of 6500/mm³. After starting prednisone, AEC improved to 200/mm^3^. The erythrocyte sedimentation rate (ESR) was within normal limits, and C-reactive protein (CRP) was 3.2 mg/dL. Antinuclear antibody (ANA) was positive at a titer of 1:640 with a homogenous pattern, while Sjögren's syndrome (SS)A and SSB were negative. Rheumatoid factor (RF), creatine kinase (CK), and aldolase were all within normal limits. The extractable nuclear antigen (ENA) panel was negative.

**Table 1:** Laboratory investigations

Laboratory values		Normal range
Absolute eosinophil count	6500/mm^3^	Less than 500/mm^3^
Erythrocyte sedimentation rate	Within normal limits	0-20 mm/hr
C-reactive protein	3.2 mg/dl	0.3-1.0 mg/dl
Antinuclear antibody	1:640 homogenous pattern	negative

 

**Figure 1:**
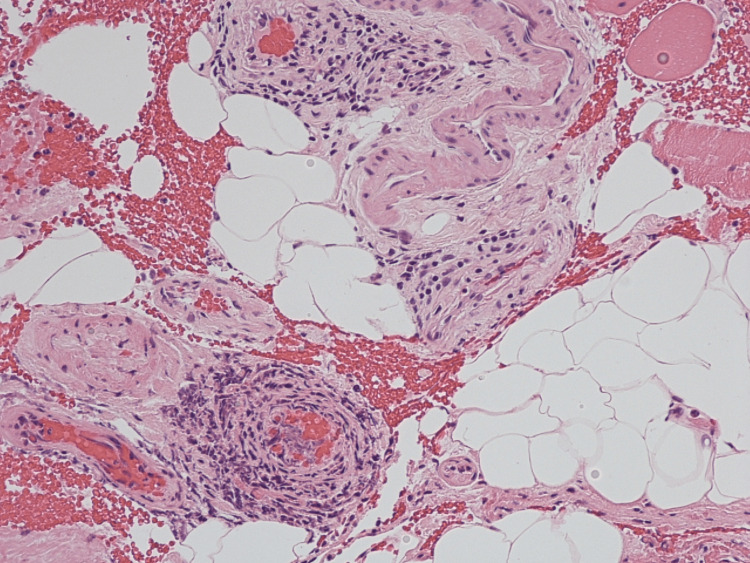
A full-thickness biopsy of the skin reveals scattered vessels with perivascular inflammation, and focally some areas of fibrous tissue demonstrate mildly increased cellularity

**Figure 2:**
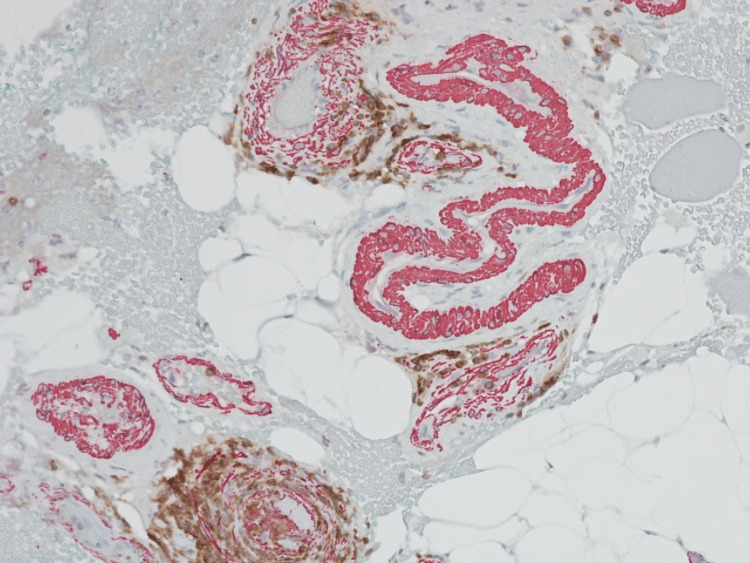
CD3/SMA staining demonstrates T cell to be involving the walls of the majority of vessels with perivascular inflammation within the muscle fascicles

The biopsy of skin muscle and fascia was done in a week, and then she was switched to prednisone 40 mg daily. Histopathologic examination results showed scattered lymphocytic infiltrate involving associated fibrous tissue (Figure 1) and perivascular lymphocytic inflammation (Figure 2) that involved vessel walls. This was because the patient had been started on prednisone prior to the biopsy. She was evaluated by hematology and had a bone marrow biopsy that showed normocellular marrow with negative JAK2 mutation cascade, negative for BCR-ABL1, with no evidence of underlying myeloproliferative disorder. Her prednisone dose was increased to 60 mg daily for two weeks, and her blood glucose was monitored. Steroids helped with swelling, but she continued to have stiffness. She was then started with methotrexate, which led to improvement in her symptoms, with softening in tissues in her arms.

## Discussion

EF is a systemic inflammatory condition that causes myalgia, soft tissue edema, skin induration, peripheral eosinophilia, hypergammaglobulinemia, and an elevated ESR. Shulman first mentioned it in 1974 [1]. It is a connective tissue condition that resembles scleroderma in several ways. The hands and fingers are typically spared from the effects of sclerodermatous alterations, which cause the skin to become stiff, wrinkled, and closely attached to the underlying fascia. Raynaud's phenomenon, telangiectasias, and extra-cutaneous symptoms are typically absent, in contrast to scleroderma [2]. Of the patients, 63-93% have peripheral blood eosinophilia, over 50% have hypergammaglobulinemia, and 26-63% have high ESR [3]. EF tissue biopsy usually shows fascial thickening with massive infiltration of inflammatory cells, such as plasma cells and neutrophils, and visible eosinophil infiltration, but this predominantly occurs in the early stages of the disease [4]. Scleroderma biopsy lesions mostly occur in the dermis and can be seen as fibrosis in the dermis, which can also involve fascia and muscle as the disease progresses [5].

Although the cause of EF is yet unknown, some potential triggers include the beginning of hemodialysis, vigorous activity, and Borrelia burgdorferi infection. Additionally, certain drug exposure has been connected. The adverse effects of simvastatin on the skin, particularly the emergence of EF, have been reported for many years [3,6].

It is important to take en bloc biopsies from the skin to the fascia. Sometimes the biopsy reveals a pronounced thickening of the fascia. Lymphocytes and plasma cells are the main inflammatory cell infiltrators visible in the hypertrophied fascia. Only evident in the early stages of the disease, eosinophilic infiltration is helpful for diagnosis. In around 50% of the cases that are investigated, the fascia has eosinophilic infiltration. The fascia is mostly affected by the lesions in this syndrome, though fibrosis can spread to the dermis and be accompanied by edema, dermal collagen fiber proliferation, and a modest infiltration of inflammatory cells, primarily lymphocytes [7].

Although eosinophilia, increased sedimentation rate, and hypergammaglobulinemia are seen, eosinophilia frequently resolves during the course of the disease or presents briefly during the early stages and then dissipates. In around 60% of cases, elevated sedimentation rate and hypergammaglobulinemia are found. Serum aldolase levels are increased in many cases, which is recognized to suggest disease activity. Serum type III procollagen peptide (PIIIP) levels reflect disease activity as well, making it an excellent marker for this condition. Antinuclear antibodies or rheumatoid factor are found in about 10% of patients [3,6,8].

Systemic glucocorticoids are the mainstay of treatment. Patients who do not respond to initial therapy with glucocorticoids or who relapse with skin involvement require additional therapy with immunosuppressive or immunomodulatory agents to obtain a therapeutic response [9].

Histologically, EF and localized scleroderma are frequently difficult to distinguish; thus, a clinical distinction is required. Localized scleroderma has fibrosis mostly in the dermis, whereas EF has fibrosis primarily in the fascia. Fibrosis can be detected not only in the dermis and subcutaneous layers of localized scleroderma lesions, but also in the underlying fascia, muscle, and bone. Furthermore, fibrosis can spread from the fascia to the dermis as EF advances. Finally, in many cases of EF, eosinophilic tissue infiltration is not found. Because of these variables, histological distinction is challenging [9].

EF is characterized by bilateral, symmetrical, and widespread swelling of the limbs, whereas localized scleroderma can be unilateral or bilateral, with a well-defined border. Although this can help with distinction, atypical forms of EF can be difficult to distinguish from linear scleroderma or some cases of subcutaneous localized scleroderma (morphea profunda) [6,10].

## Conclusions

EF is a rare condition that can mimic systemic sclerosis but presents with distinct clinical features. The patient's presentation in the current report was highly suspicious for EF, given the rapid onset of skin tightening, peripheral eosinophilia, and negative RF with negative systemic sclerosis findings including absence of sclerodactyly, Raynaud's phenomenon, and skin tightening in the hands and face, which differentiates EF. A biopsy of the affected area is essential for confirmation. Careful monitoring and management of comorbid conditions such as diabetes mellitus are essential during high-dose steroid therapy.
